# HOXB9 promotes endometrial cancer progression by targeting E2F3

**DOI:** 10.1038/s41419-018-0556-3

**Published:** 2018-05-03

**Authors:** Junhu Wan, Hongyang Liu, Quanling Feng, Jun Liu, Liang Ming

**Affiliations:** 1 Department of Clinical Laboratory, The First Affiliated Hospital of Zhengzhou University, 450052 Zhengzhou, Henan China; 2 Department of Obstetrics and Gynecology, The Third Affiliated Hospital of Zhengzhou University, 450052 Zhengzhou, Henan China; 3 Department of General Surgery, Zhecheng People’s Hospital, 476000 Shangqiu, Henan China

## Abstract

HOXB9, as a HOX family transcription factor, playing a significant role in embryonic development and cancer progression. However, the function of HOXB9 and its precise mechanism in regulating endometrial cancer progression remains unknown. Here, we demonstrated that the expression of HOXB9 was increased in endometrial cancer, and associated with histological grade and lymph node metastasis. In addition, elevated HOXB9 predicts a poor prognosis in endometrial cancer patients. Interestingly, bioinformatics analysis of TCGA cancer database showed that HOXB9 expression is positively correlated with E2F3 expression. Moreover, HOXB9 promoted E2F3 expression by directly targeting to its promoter. Furthermore, we found that knocking down E2F3 abolished the ability of HOXB9 in enhancing cell migration. Taken together, for the first, we demonstrated the function and mechanism of HOXB9 in regulating endometrial cancer progression, and indicated HOXB9 may be a novel prognostic marker of endometrial cancer.

## Introduction

Endometrial cancer (EC) is one of the leading causes of gynecologic malignancies^1,2^. The patients present with high-grade tumors are aggressive, and frequently diagnosed with tumors spread beyond the uterus^3,4^. Surgery is the major treatment for EC, while it is quite important to develop new therapeutic strategies for EC^5^. The investigation of new molecular mechanisms in EC may be useful to identify new diagnostic and therapeutic targets.

The HOX genes, encode a group of transcription factors, share a highly conserved homeobox domain. In vertebrates, the whole 39 HOX genes are identified and grouped into four clusters (HOXA, B, C and D)^6–8^. During embryogenesis, sequential HOX expression from 3ʹ to 5ʹ along the anterior­posterior (AP) axis could control body segmentation according to the rules of spatial and temporal collinearity^8–11^. In particular, HOXB9 together with other HOX genes controls the specification of thoracic skeletal elements and mammary gland development^12,13^.

In addition to its key roles in embryo development, HOXB9 was also found to be participated in the regulation of numerous human cancers^14–17^. In the previous study, we found HOXB9-regulated lung adenocarcinoma progression by directly targeting JMJD6^18^. In addition, upregulation of HOXB9 showed poor overall survival in invasive human breast cancer, and promoted epithelial-to-mesenchymal transition (EMT)^19–21^. However, increased expression of HOXB9 showed better overall survival in colon adenocarcinoma, pancreatic ductal adenocarcinoma and gastric carcinoma patients^15–17^, indicating HOXB9 played an opposite role in these cancer progression.

The E2F family of transcription factors is conserved from nematodes to mammals, which were identified as factors that control the cell cycle, differentiation, apoptosis and stress responses^22,23^. As an important member of E2F family, E2F3, encodes two isoforms, E2f3a and E2f3b, whose expression is driven by distinct promoters^24^. Overexpressed E2F3 expression has been reported in ovarian, prostate and lung cancers^25–27^. In the thyroid cancer mouse model, a role for E2F3 has been found in the metastasis of tumor cells to the liver and lung^28^. Using conditional knockout approaches, Trikha et al. reported that ablation of E2F3 in tumor-associated macrophages (TAMs) results in decreased pulmonary metastasis without affecting the tumor growth^29^. Tsuruta et al. reported that the expression of E2F3 was upregulated in 28.6% (20 of 70) of EC cases^30^. However, the role of E2F3 in EC progression remains unclear.

Although HOXB9 has been extensively investigated in some cancer types, its role in EC has never been reported. In this report, we demonstrated that HOXB9 is overexpressed in EC, and is correlated with EC cell migration. Moreover, HOXB9 promotes EC progression by targeting oncogenic protein E2F3.

## Results

### The expression of HOXB9 and its clinical significance in EC

**Fig. 1:**
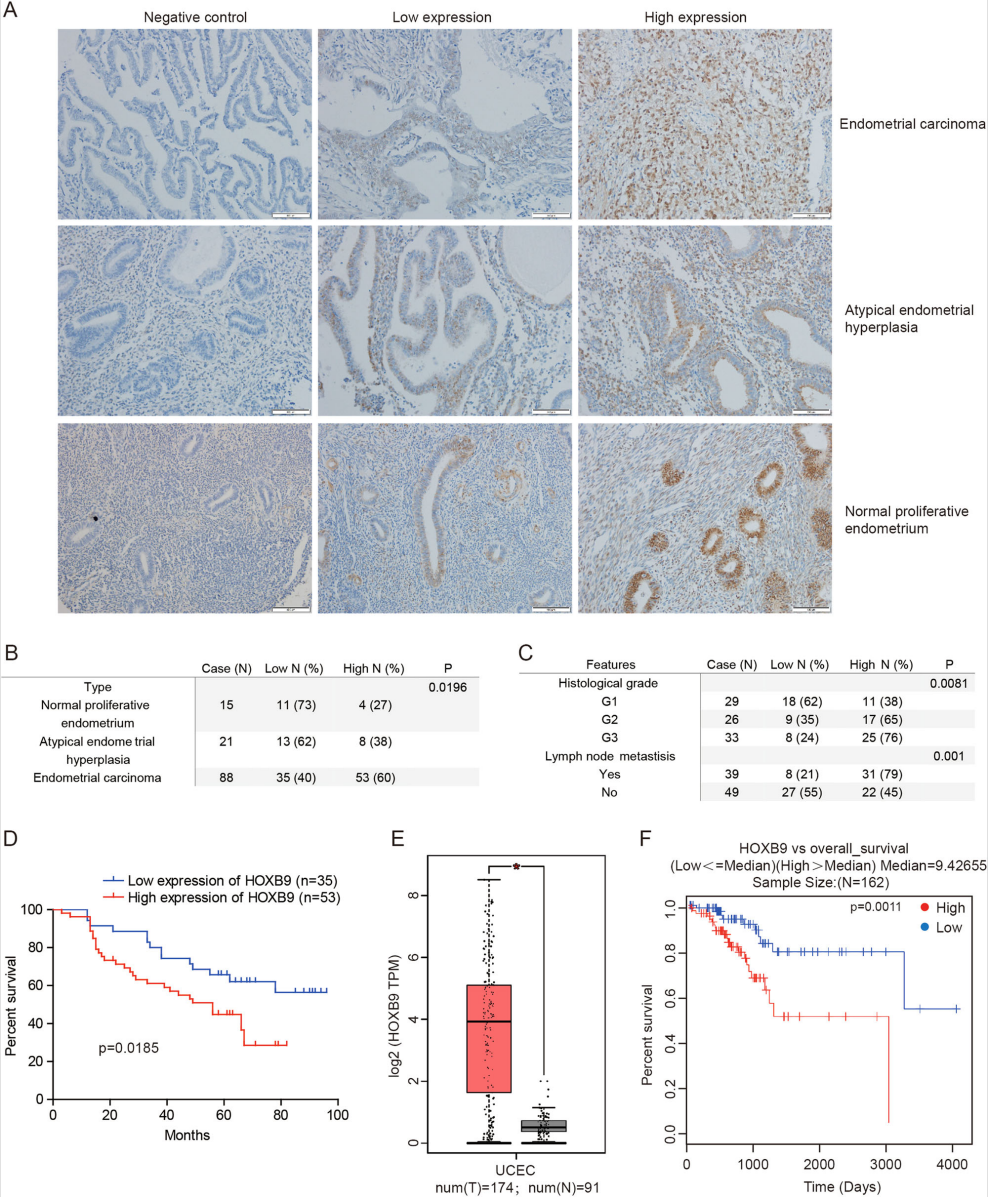
The expression of HOXB9 and its clinical significance in endometrial cancer. **a** The staining scores of HOXB9 expression level were dichotomized into two groups, low (score of 0 or 1) and high (score of 2 or 3). We have displayed the representative images of “low” and “high” staining, as well as negative controls, in endometrial carcinoma, normal proliferative endometrium and atypical hyperplasia tissues. **b** The high expression ratio of HOXB9 in normal proliferative endometrium, atypical endometrial hyperplasia and endometrial carcinoma were showed. **c** The HOXB9 expression correlation in endometrial carcinoma with histological grade and lymph node metastasis status. **d** The Kaplan–Meier analysis of HOXB9 expression in endometrial carcinoma was performed. **e, f** The expression level of HOXB9 in human endometrial cancer compared with normal tissues in TCGA database **e**, and the Kaplan–Meier analysis of HOXB9 expression in human endometrial cancer in TCGA **f**. The statistical analyses were performed by one-way ANOVA analyses **b, c**, Student’s *t*-test **e** and Kaplan–Meier analysis **d, f**. *P < 0.05

To determine the expression of HOXB9 in EC, we analyzed its expression in a series of 88 endometrial carcinoma, 15 normal proliferative endometrium and 21 atypical endometrial hyperplasia by immunohistochemistry. The expression of HOXB9 was observed mainly in the nucleus of cells. The staining scores of HOXB9 expression level were dichotomized into two groups, low (score of 0 or 1) and high (score of 2 or 3). The representative images of “low” and “high” staining, as well as negative controls, in endometrial carcinoma, normal proliferative endometrium and atypical hyperplasia tissues were shown in Fig. 1a. We found that the high expression ratio and expression level of HOXB9 in normal proliferative endometrium, atypical endometrial hyperplasia and endometrial carcinoma were gradually increased (*P* = 0.0196; Fig. 1b and S1a). The correlations between HOXB9 expression and clinicopathologic characteristics of endometrial carcinoma are shown in Table 1. In endometrial carcinoma, HOXB9 expression correlated only with histological grade (*P* = 0.0081) and lymph node metastasis status (*P* = 0.001; Fig. 1c). The HOXB9 expression score in G1, G2 and G3 was gradually increased (Fig. S1b) and tumors with metastasis expressed significantly higher levels than tumors without metastasis (Fig. S1c). However, no correlation between HOXB9 expression with patients’ age, surgical staging and muscular invasion endomembrane (Table 1). In addition, we found high HOXB9 expression level correlated significantly with shorter disease-specific survival time as illustrated in Fig. 1d.

**Table 1:** Relationship between HOXB9 expression and clinicopathological features in endometrial carcinomas

	HOXB9			
Features	Case (*N*)	Low *N* (%)	High *N* (%)	*P*-value
Age				0.2396
≤50	41	19 (46)	22 (54)	
>50	47	16 (34)	31 (66)	
Surgical staging				0.1041
I	53	19 (36)	34 (64)	
II	18	11 (61)	7 (39)	
III–IV	17	5 (29)	12 (71)	
Histological grade				0.0081
G1	29	18 (62)	11 (38)	
G2	26	9 (35)	17 (65)	
G3	33	8 (24)	25 (76)	
Muscular invasion endomembrane				0.2446
≤1/2	39	12 (31)	27 (69)	
>1/2	49	21 (43)	28 (57)	
Lymph node metastisis				0.001
Yes	39	8 (21)	31 (79)	
No	49	27 (55)	22 (45)	

Besides, the results of TCGA database, which were mainly analyzed by the web-based tools, Gene Expression Profiling Interactive Analysis (GEPIA, http://gepia.cancer-pku.cn/)^31^ and the LinkedOmics database (http://www.linkedomics.org)^32^, showed that HOXB9 expression was significantly overexpressed in human EC tissues compared with normal tissues (Fig. 1e and Table 2), and elevated HOXB9 expression level corresponds to a shorter overall survival in EC (Fig. 1f). These findings suggested that HOXB9 is highly expressed in EC and elevated HOXB9 may predict a poor outcome for EC patients.

**Table 2:** The sample information on TCGA database for endometrial carcinoma

Sample ID	Sample_type	Clinical_stage	_OS	Histological_type
TCGA-AX-A05S-01	Primary tumor	Stage IIIC	413	Endometrioid endometrial adenocarcinoma
TCGA-AP-A05P-01	Primary tumor	Stage IA	636	Endometrioid endometrial adenocarcinoma
TCGA-AP-A05O-01	Primary tumor	Stage IB	384	Endometrioid endometrial adenocarcinoma
TCGA-AP-A05N-01	Primary tumor	Stage IA	726	Endometrioid endometrial adenocarcinoma
TCGA-DI-A2QU-11	Primary tumor	Stage IVB	946	Endometrioid endometrial adenocarcinoma
TCGA-AJ-A2QN-01	Primary tumor	Stage II	1069	Endometrioid endometrial adenocarcinoma
TCGA-AJ-A2QL-11	Primary tumor	Stage IIIC1	602	Endometrioid endometrial adenocarcinoma
TCGA-AJ-A2QK-01	Primary tumor	Stage IA	643	Endometrioid endometrial adenocarcinoma
TCGA-EY-A2OM-01	Primary tumor	Stage IA	694	Endometrioid endometrial adenocarcinoma
TCGA-BG-A2L7-11	Primary tumor	Stage IA	612	Endometrioid endometrial adenocarcinoma
TCGA-DF-A2KS-01	Primary tumor	Stage IB	3318	Endometrioid endometrial adenocarcinoma
TCGA-AX-A2HK-01	Primary tumor	Stage IIIC	90	Endometrioid endometrial adenocarcinoma
TCGA-AX-A2HJ-01	Primary tumor	Stage IC	935	Endometrioid endometrial adenocarcinoma
TCGA-AX-A2HG-01	Primary tumor	Stage IC	1163	Endometrioid endometrial adenocarcinoma
TCGA-AX-A2HD-01	Primary tumor	Stage IIIA	1414	Endometrioid endometrial adenocarcinoma
TCGA-AX-A2HC-01	Primary tumor	Stage IIIC	1044	Endometrioid endometrial adenocarcinoma
TCGA-AX-A2HA-11	Primary tumor	Stage IB	2475	Endometrioid endometrial adenocarcinoma
TCGA-AX-A2H8-11	Primary tumor	Stage IB	1861	Endometrioid endometrial adenocarcinoma
TCGA-AX-A2H7-11	Primary tumor	Stage IC	1241	Endometrioid endometrial adenocarcinoma
TCGA-D1-A2G6-01	Primary tumor	Stage IIIC	258	Endometrioid endometrial adenocarcinoma
TCGA-D1-A2G5-01	Primary tumor	Stage IB	1455	Endometrioid endometrial adenocarcinoma
TCGA-FI-A2F9-01	Primary tumor	Stage IA	1803	Endometrioid endometrial adenocarcinoma
TCGA-FI-A2F4-01	Primary tumor	Stage IC	2230	Endometrioid endometrial adenocarcinoma
TCGA-FI-A2D6-01	Primary tumor	Stage IB	767	Endometrioid endometrial adenocarcinoma
TCGA-FI-A2D5-01	Primary tumor	Stage IVB	421	Endometrioid endometrial adenocarcinoma
TCGA-FI-A2D4-01	Primary tumor	Stage IIIC	243	Endometrioid endometrial adenocarcinoma
TCGA-FI-A2D0-01	Primary tumor	Stage IA	1023	Endometrioid endometrial adenocarcinoma
TCGA-FI-A2CX-01	Primary tumor	Stage IB	2031	Endometrioid endometrial adenocarcinoma
TCGA-BG-A2AE-11	Primary tumor	Stage IB	721	Endometrioid endometrial adenocarcinoma
TCGA-BG-A2AD-01	Primary tumor	Stage IIIB	609	Endometrioid endometrial adenocarcinoma
TCGA-EC-A1QX-01	Primary tumor	Stage IB	224	Endometrioid endometrial adenocarcinoma
TCGA-AW-A1PO-01	Primary tumor	Stage IIIA	17	Endometrioid endometrial adenocarcinoma
TCGA-A5-A1OK-01	Primary tumor	Stage IIIA	917	Endometrioid endometrial adenocarcinoma
TCGA-A5-A1OJ-01	Primary tumor	Stage IA	1006	Endometrioid endometrial adenocarcinoma
TCGA-D1-A1O8-01	Primary tumor	Stage IIIC	120	Endometrioid endometrial adenocarcinoma
TCGA-D1-A1O7-01	Primary tumor	Stage IA	32	Endometrioid endometrial adenocarcinoma
TCGA-D1-A1O5-01	Primary tumor	Stage IA	458	Endometrioid endometrial adenocarcinoma
TCGA-D1-A1O0-01	Primary tumor	Stage IA	63	Endometrioid endometrial adenocarcinoma
TCGA-D1-A1NZ-01	Primary tumor	Stage IA	548	Endometrioid endometrial adenocarcinoma
TCGA-D1-A1NY-01	Primary tumor	Stage IB	513	Endometrioid endometrial adenocarcinoma
TCGA-D1-A1NS-01	Primary tumor	Stage IA	43	Endometrioid endometrial adenocarcinoma
TCGA-DI-A1NO-11	Primary tumor	Stage IIIA	285	Endometrioid endometrial adenocarcinoma
TCGA-EC-A1NJ-01	Primary tumor	Stage IA	488	Endometrioid endometrial adenocarcinoma
TCGA-B5-A1MZ-01	Primary tumor	Stage IA	1598	Endometrioid endometrial adenocarcinoma
TCGA-B5-A1MX-01	Primary tumor	Stage IB	1473	Endometrioid endometrial adenocarcinoma
TCGA-B5-A1MV-01	Primary tumor	Stage IA	1565	Endometrioid endometrial adenocarcinoma
TCGA-B5-A1MR-01	Primary tumor	Stage IIIA	6859	Endometrioid endometrial adenocarcinoma
TCGA-E6-A1M0-11	Primary tumor	Stage IIIC	1793	Endometrioid endometrial adenocarcinoma
TCGA-E6-A1LX-01	Primary tumor	Stage IB	711	Endometrioid endometrial adenocarcinoma
TCGA-EY-A1H0-01	Primary tumor	Stage IIIC2	588	Endometrioid endometrial adenocarcinoma
TCGA-EY-A1GW-01	Primary tumor	Stage IVB	337	Endometrioid endometrial adenocarcinoma
TCGA-EY-A1GU-01	Primary tumor	Stage IB	997	Endometrioid endometrial adenocarcinoma
TCGA-EY-A1GT-01	Primary tumor	Stage IIIA	490	Endometrioid endometrial adenocarcinoma
TCGA-EY-A1GR-01	Primary tumor	Stage IA	536	Endometrioid endometrial adenocarcinoma
TCGA-EY-A1GQ-01	Primary tumor	Stage IB	544	Endometrioid endometrial adenocarcinoma
TCGA-EY-A1GK-01	Primary tumor	Stage IB	693	Endometrioid endometrial adenocarcinoma
TCGA-EY-A1GJ-01	Primary tumor	Stage IA	727	Endometrioid endometrial adenocarcinoma
TCGA-EY-A1GI-01	Primary tumor	Stage IB	710	Endometrioid endometrial adenocarcinoma
TCGA-EY-A1GH-01	Primary tumor	Stage IA	710	Endometrioid endometrial adenocarcinoma
TCGA-EY-A1GF-01	Primary tumor	Stage IA	826	Endometrioid endometrial adenocarcinoma
TCGA-EY-A1GE-01	Primary tumor	Stage IA	741	Endometrioid endometrial adenocarcinoma
TCGA-EY-A1GD-01	Primary tumor	Stage IA	1639	Endometrioid endometrial adenocarcinoma
TCGA-EY-A1GC-01	Primary tumor	Stage IB	1647	Endometrioid endometrial adenocarcinoma
TCGA-EY-A1G8-01	Primary tumor	Stage IB	456	Endometrioid endometrial adenocarcinoma
TCGA-EY-A1G7-01	Primary tumor	Stage II	189	Endometrioid endometrial adenocarcinoma
TCGA-AP-A1E4-01	Primary tumor	Stage IB	1555	Endometrioid endometrial adenocarcinoma
TCGA-AP-A1E3-01	Primary tumor	Stage IIIC1	847	Endometrioid endometrial adenocarcinoma
TCGA-AP-A1E1-01	Primary tumor	Stage IA	1395	Endometrioid endometrial adenocarcinoma
TCGA-AP-A1E0-01	Primary tumor	Stage IIIC2	1832	Endometrioid endometrial adenocarcinoma
TCGA-AP-A1DV-01	Primary tumor	Stage II	204	Endometrioid endometrial adenocarcinoma
TCGA-AP-A1DR-01	Primary tumor	Stage IIIC1	1495	Endometrioid endometrial adenocarcinoma
TCGA-AP-A1DP-01	Primary tumor	Stage IA	1081	Endometrioid endometrial adenocarcinoma
TCGA-AP-A1DO-01	Primary tumor	Stage IA	952	Endometrioid endometrial adenocarcinoma
TCGA-AP-A1DM-01	Primary tumor	Stage IA	1700	Endometrioid endometrial adenocarcinoma
TCGA-AP-A1DK-01	Primary tumor	Stage IA	2767	Endometrioid endometrial adenocarcinoma
TCGA-AP-A1DH-01	Primary tumor	Stage IA	2180	Endometrioid endometrial adenocarcinoma
TCGA-AX-A1CN-01	Primary tumor	Stage IIB	1143	Endometrioid endometrial adenocarcinoma
TCGA-AX-A1CK-11	Primary tumor	Stage IB	2441	Endometrioid endometrial adenocarcinoma
TCGA-AX-A1CJ-11	Primary tumor	Stage IC	2233	Endometrioid endometrial adenocarcinoma
TCGA-AX-A1CI-01	Primary tumor	Stage IB	2600	Endometrioid endometrial adenocarcinoma
TCGA-AX-A1CF-01	Primary tumor	Stage IVB	2337	Endometrioid endometrial adenocarcinoma
TCGA-AX-A1CE-01	Primary tumor	Stage IB	1037	Endometrioid endometrial adenocarcinoma
TCGA-AX-A1C9-01	Primary tumor	Stage IB	802	Endometrioid endometrial adenocarcinoma
TCGA-AX-A1C5-01	Primary tumor	Stage IIIC	1001	Endometrioid endometrial adenocarcinoma
TCGA-AX-A1C4-01	Primary tumor	Stage IB	404	Endometrioid endometrial adenocarcinoma
TCGA-DI-A1BY-01	Primary tumor	Stage IIB	1114	Endometrioid endometrial adenocarcinoma
TCGA-D1-A0ZV-01	Primary tumor	Stage IB	728	Endometrioid endometrial adenocarcinoma
TCGA-D1-A0ZU-01	Primary tumor	Stage IB	872	Endometrioid endometrial adenocarcinoma
TCGA-D1-A0ZS-01	Primary tumor	Stage IB	148	Endometrioid endometrial adenocarcinoma
TCGA-D1-A0ZR-01	Primary tumor	Stage IB	17	Endometrioid endometrial adenocarcinoma
TCGA-D1-A0ZQ-01	Primary tumor	Stage IB	260	Endometrioid endometrial adenocarcinoma
TCGA-D1-A0ZO-01	Primary tumor	Stage IB	602	Endometrioid endometrial adenocarcinoma
TCGA-D1-A0ZN-01	Primary tumor	Stage IA	512	Endometrioid endometrial adenocarcinoma
TCGA-BG-A0YU-01	Primary tumor	Stage IA	451	Endometrioid endometrial adenocarcinoma
TCGA-BS-A0WQ-01	Primary tumor	Stage IA	2022	Endometrioid endometrial adenocarcinoma
TCGA-DI-A0WH-01	Primary tumor	Stage IB	496	Endometrioid endometrial adenocarcinoma
TCGA-BG-A0W2-01	Primary tumor	Stage IA	1721	Endometrioid endometrial adenocarcinoma
TCGA-BG-A0W1-01	Primary tumor	Stage II	1607	Endometrioid endometrial adenocarcinoma
TCGA-BG-A0VZ-01	Primary tumor	Stage IIIA	1601	Endometrioid endometrial adenocarcinoma
TCGA-BG-A0VX-01	Primary tumor	Stage IB	1043	Endometrioid endometrial adenocarcinoma
TCGA-BG-A0VW-01	Primary tumor	Stage IA	1582	Endometrioid endometrial adenocarcinoma
TCGA-BG-A0VV-01	Primary tumor	Stage IA	1553	Endometrioid endometrial adenocarcinoma
TCGA-BG-A0VT-01	Primary tumor	Stage IIIA	1568	Endometrioid endometrial adenocarcinoma
TCGA-A5-A0VQ-01	Primary tumor	Stage IA	485	Endometrioid endometrial adenocarcinoma
TCGA-A5-A0VP-01	Primary tumor	Stage IA	1288	Endometrioid endometrial adenocarcinoma
TCGA-A5-A0VO-01	Primary tumor	Stage IB	875	Endometrioid endometrial adenocarcinoma
TCGA-BS-A0VI-01	Primary tumor	Stage IIB	2646	Endometrioid endometrial adenocarcinoma
TCGA-BS-A0V8-01	Primary tumor	Stage IIB	2544	Endometrioid endometrial adenocarcinoma
TCGA-BS-A0V6-01	Primary tumor	Stage IB	2725	Endometrioid endometrial adenocarcinoma
TCGA-BS-A0UV-01	Primary tumor	Stage IIIC	2228	Endometrioid endometrial adenocarcinoma
TCGA-BS-A0UT-01	Primary tumor	Stage IIB	2212	Endometrioid endometrial adenocarcinoma
TCGA-BS-A0UM-01	Primary tumor	Stage IB	2644	Endometrioid endometrial adenocarcinoma
TCGA-BS-A0UL-01	Primary tumor	Stage IA	2481	Endometrioid endometrial adenocarcinoma
TCGA-BS-A0UJ-01	Primary tumor	Stage IA	2506	Endometrioid endometrial adenocarcinoma
TCGA-BS-A0UF-01	Primary tumor	Stage IB	2611	Endometrioid endometrial adenocarcinoma
TCGA-BS-A0UA-01	Primary tumor	Stage IB	3495	Endometrioid endometrial adenocarcinoma
TCGA-BS-A0U9-01	Primary tumor	Stage IIA	2825	Endometrioid endometrial adenocarcinoma
TCGA-BS-A0U8-01	Primary tumor	Stage IIIC	2963	Endometrioid endometrial adenocarcinoma
TCGA-BS-A0U7-01	Primary tumor	Stage IC	935	Endometrioid endometrial adenocarcinoma
TCGA-BS-A0U5-01	Primary tumor	Stage IA	2935	Endometrioid endometrial adenocarcinoma
TCGA-BS-A0TJ-01	Primary tumor	Stage IC	2068	Endometrioid endometrial adenocarcinoma
TCGA-BS-A0TI-01	Primary tumor	Stage IC	1882	Endometrioid endometrial adenocarcinoma
TCGA-BS-A0TG-01	Primary tumor	Stage IIIA	2371	Endometrioid endometrial adenocarcinoma
TCGA-BS-A0TE-01	Primary tumor	Stage IVB	146	Endometrioid endometrial adenocarcinoma
TCGA-BS-A0TD-01	Primary tumor	Stage IB	2379	Endometrioid endometrial adenocarcinoma
TCGA-BS-A0TC-01	Primary tumor	Stage IB	2602	Endometrioid endometrial adenocarcinoma
TCGA-BS-A0TA-01	Primary tumor	Stage IVB	740	Endometrioid endometrial adenocarcinoma
TCGA-BS-A0T9-01	Primary tumor	Stage IVB	1428	Endometrioid endometrial adenocarcinoma
TCGA-BG-A0RY-01	Primary tumor	Stage IB	469	Endometrioid endometrial adenocarcinoma
TCGA-A5-A0RA-01	Primary tumor	Stage IB	884	Endometrioid endometrial adenocarcinoma
TCGA-A5-A0R9-01	Primary tumor	Stage IA	749	Endometrioid endometrial adenocarcinoma
TCGA-A5-A0R8-01	Primary tumor	Stage IB	596	Endometrioid endometrial adenocarcinoma
TCGA-A5-A0R7-01	Primary tumor	Stage IA	535	Endometrioid endometrial adenocarcinoma
TCGA-BG-A0MU-01	Primary tumor	Stage IIIA	617	Endometrioid endometrial adenocarcinoma
TCGA-BG-A0MT-01	Primary tumor	Stage IA	644	Endometrioid endometrial adenocarcinoma
TCGA-BG-A0MS-01	Primary tumor	Stage IIIC1	1882	Endometrioid endometrial adenocarcinoma
TCGA-BG-A0MQ-01	Primary tumor	Stage IA	1817	Endometrioid endometrial adenocarcinoma
TCGA-BG-A0MO-01	Primary tumor	Stage IA	1309	Endometrioid endometrial adenocarcinoma
TCGA-BG-A0MI-01	Primary tumor	Stage IA	714	Endometrioid endometrial adenocarcinoma
TCGA-BG-A0MH-01	Primary tumor	Stage IC	1930	Endometrioid endometrial adenocarcinoma
TCGA-BG-A0MG-01	Primary tumor	Stage IA	1477	Endometrioid endometrial adenocarcinoma
TCGA-BG-A0MC-01	Primary tumor	Stage IA	1263	Endometrioid endometrial adenocarcinoma
TCGA-BG-A0MA-11	Primary tumor	Stage IB	326	Endometrioid endometrial adenocarcinoma
TCGA-BG-A0M9-01	Primary tumor	Stage IB	2270	Endometrioid endometrial adenocarcinoma
TCGA-BG-A0M8-01	Primary tumor	Stage IA	2020	Endometrioid endometrial adenocarcinoma
TCGA-BG-A0M7-01	Primary tumor	Stage IIIC1	1937	Endometrioid endometrial adenocarcinoma
TCGA-BG-A0M4-01	Primary tumor	Stage IA	2167	Endometrioid endometrial adenocarcinoma
TCGA-BG-A0M3-01	Primary tumor	Stage IB	1071	Endometrioid endometrial adenocarcinoma
TCGA-BG-A0M2-01	Primary tumor	Stage IB	637	Endometrioid endometrial adenocarcinoma
TCGA-BG-A0M0-01	Primary tumor	Stage IA	588	Endometrioid endometrial adenocarcinoma
TCGA-BG-A0LX-01	Primary tumor	Stage IB	614	Endometrioid endometrial adenocarcinoma
TCGA-BG-A0LW-01	Primary tumor	Stage IA	566	Endometrioid endometrial adenocarcinoma
TCGA-AP-A0LV-01	Primary tumor	Stage IA	763	Endometrioid endometrial adenocarcinoma
TCGA-AP-A0LT-01	Primary tumor	Stage II	1497	Endometrioid endometrial adenocarcinoma
TCGA-AP-A0LS-01	Primary tumor	Stage IA	2904	Endometrioid endometrial adenocarcinoma
TCGA-AP-A0LQ-01	Primary tumor	Stage IA	2457	Endometrioid endometrial adenocarcinoma
TCGA-AP-A0LP-01	Primary tumor	Stage IA	1875	Endometrioid endometrial adenocarcinoma
TCGA-AP-A0LO-01	Primary tumor	Stage IA	1016	Endometrioid endometrial adenocarcinoma
TCGA-AP-A0LN-01	Primary tumor	Stage IA	2510	Endometrioid endometrial adenocarcinoma
TCGA-AP-A0LM-01	Primary tumor	Stage IIIC2	825	Endometrioid endometrial adenocarcinoma
TCGA-AP-A0LL-01	Primary tumor	Stage IA	2554	Endometrioid endometrial adenocarcinoma
TCGA-AP-A0LJ-01	Primary tumor	Stage IA	1421	Endometrioid endometrial adenocarcinoma
TCGA-AP-A0LG-01	Primary tumor	Stage IA	2015	Endometrioid endometrial adenocarcinoma
TCGA-AP-A0LF-01	Primary tumor	Stage IVB	2859	Endometrioid endometrial adenocarcinoma
TCGA-AP-A0LE-01	Primary tumor	Stage IA	3357	Endometrioid endometrial adenocarcinoma
TCGA-AP-A0LD-01	Primary tumor	Stage IB	3589	Endometrioid endometrial adenocarcinoma
TCGA-B5-A0KB-01	Primary tumor	Stage IB	824	Endometrioid endometrial adenocarcinoma
TCGA-B5-A0K7-01	Primary tumor	Stage IA	1933	Endometrioid endometrial adenocarcinoma
TCGA-B5-A0K6-01	Primary tumor	Stage IA	1953	Endometrioid endometrial adenocarcinoma
TCGA-B5-A0K4-01	Primary tumor	Stage IB	1757	Endometrioid endometrial adenocarcinoma
TCGA-B5-A0K3-01	Primary tumor	Stage IA	700	Endometrioid endometrial adenocarcinoma
TCGA-B5-A0K2-01	Primary tumor	Stage IIIA	2209	Endometrioid endometrial adenocarcinoma
TCGA-B5-A0K1-01	Primary tumor	Stage IA	1110	Endometrioid endometrial adenocarcinoma
TCGA-B5-A0K0-01	Primary tumor	Stage IA	1478	Endometrioid endometrial adenocarcinoma
TCGA-FL-A1YN-11	Solid tissue normal			
TCGA-FL-A1YU-11	Solid tissue normal			
TCGA-FL-A1YL-11	Solid tissue normal			
TCGA-FL-A1YI-11	Solid tissue normal			
TCGA-AX-A0J0-11	Solid tissue normal			
TCGA-FL-A1YQ-11	Solid tissue normal			
TCGA-FL-A1YF-11	Solid tissue normal			
TCGA-FL-A3WE-11	Solid tissue normal			
TCGA-FL-A1YV-11	Solid tissue normal			
TCGA-FL-A1YT-11	Solid tissue normal			
TCGA-AJ-A3NC-11	Solid tissue normal			
TCGA-AJ-A3NE-11	Solid tissue normal			
TCGA-AX-A2HC-11	Solid tissue normal			
TCGA-AX-A2HD-11	Solid tissue normal			
TCGA-BK-A4ZD-11	Solid tissue normal			
TCGA-BG-A3EW-11	Solid tissue normal			
TCGA-FL-A1YH-11	Solid tissue normal			
TCGA-AJ-A3NH-11	Solid tissue normal			
TCGA-AX-A05Y-11	Solid tissue normal			
TCGA-DI-A2QY-11	Solid tissue normal			
TCGA-BG-A2AD-11	Solid tissue normal			
TCGA-FL-A1YG-11	Solid tissue normal			
TCGA-AX-A0IZ-11	Solid tissue normal			
TCGA-BG-A3PP-11	Solid tissue normal			
TCGA-AX-A2H5-01	Solid tissue normal			
TCGA-AX-A2H4-11	Solid tissue normal			
TCGA-DI-A1NN-01	Solid tissue normal			
TCGA-EO-A22T-01	Solid tissue normal			
TCGA-EO-A22S-01	Solid tissue normal			
TCGA-EO-A22R-01	Solid tissue normal			
TCGA-BK-A13C-01	Solid tissue normal			
TCGA-DI-A2QU-01	Solid tissue normal			
TCGA-AJ-A2QL-01	Solid tissue normal			
TCGA-BG-A2L7-01	Solid tissue normal			
TCGA-AX-A2HA-01	Solid tissue normal			
TCGA-AX-A2H8-01	Solid tissue normal			
TCGA-AX-A2H7-01	Solid tissue normal			
TCGA-BG-A2AE-01	Solid tissue normal			
TCGA-DI-A1NO-01	Solid tissue normal			
TCGA-E6-A1M0-01	Solid tissue normal			
TCGA-AX-A1CK-01	Solid tissue normal			
TCGA-AX-A1CJ-01	Solid tissue normal			
TCGA-AX-A1CI-11	Solid tissue normal			
TCGA-AX-A1CF-11	Solid tissue normal			
TCGA-BG-A0MA-01	Solid tissue normal			
TCGA-BK-A0CB-01	Solid tissue normal			
TCGA-FL-A1YM-11	Solid tissue normal			
TCGA-FL-A1YN-11	Solid tissue normal			
TCGA-FL-A1YU-11	Solid tissue normal			
TCGA-FL-A1YL-11	Solid tissue normal			
TCGA-FL-A1YI-11	Solid tissue normal			
TCGA-AX-A0J0-11	Solid tissue normal			
TCGA-FL-A1YQ-11	Solid tissue normal			
TCGA-FL-A1YF-11	Solid tissue normal			
TCGA-FL-A3WE-11	Solid tissue normal			
TCGA-FL-A1YV-11	Solid tissue normal			
TCGA-FL-A1YT-11	Solid tissue normal			
TCGA-AJ-A3NC-11	Solid tissue normal			
TCGA-AJ-A3NE-11	Solid tissue normal			
TCGA-AX-A2HC-11	Solid tissue normal			
TCGA-AX-A2HD-11	Solid tissue normal			
TCGA-BK-A4ZD-11	Solid tissue normal			
TCGA-BG-A3EW-11	Solid tissue normal			
TCGA-FL-A1YH-11	Solid tissue normal			
TCGA-AJ-A3NH-11	Solid tissue normal			
TCGA-AX-A05Y-11	Solid tissue normal			
TCGA-DI-A2QY-11	Solid tissue normal			
TCGA-BG-A2AD-11	Solid tissue normal			
TCGA-FL-A1YG-11	Solid tissue normal			
TCGA-AX-A0IZ-11	Solid tissue normal			
TCGA-BG-A3PP-11	Solid tissue normal			
TCGA-AX-A2H5-01	Solid tissue normal			
TCGA-AX-A2H4-11	Solid tissue normal			
TCGA-DI-A1NN-01	Solid tissue normal			
TCGA-EO-A22T-01	Solid tissue normal			
TCGA-EO-A22S-01	Solid tissue normal			
TCGA-EO-A22R-01	Solid tissue normal			
TCGA-BK-A13C-01	Solid tissue normal			
TCGA-DI-A2QU-01	Solid tissue normal			
TCGA-AJ-A2QL-01	Solid tissue normal			
TCGA-BG-A2L7-01	Solid tissue normal			
TCGA-AX-A2HA-01	Solid tissue normal			
TCGA-AX-A2H8-01	Solid tissue normal			
TCGA-AX-A2H7-01	Solid tissue normal			
TCGA-BG-A2AE-01	Solid tissue normal			
TCGA-DI-A1NO-01	Solid tissue normal			
TCGA-E6-A1M0-01	Solid tissue normal			
TCGA-AX-A1CK-01	Solid tissue normal			
TCGA-AX-A1CJ-01	Solid tissue normal			
TCGA-AX-A1CI-11	Solid tissue normal			
TCGA-AX-A1CF-11	Solid tissue normal			

### HOXB9 promotes EC cell migration

**Fig. 2:**
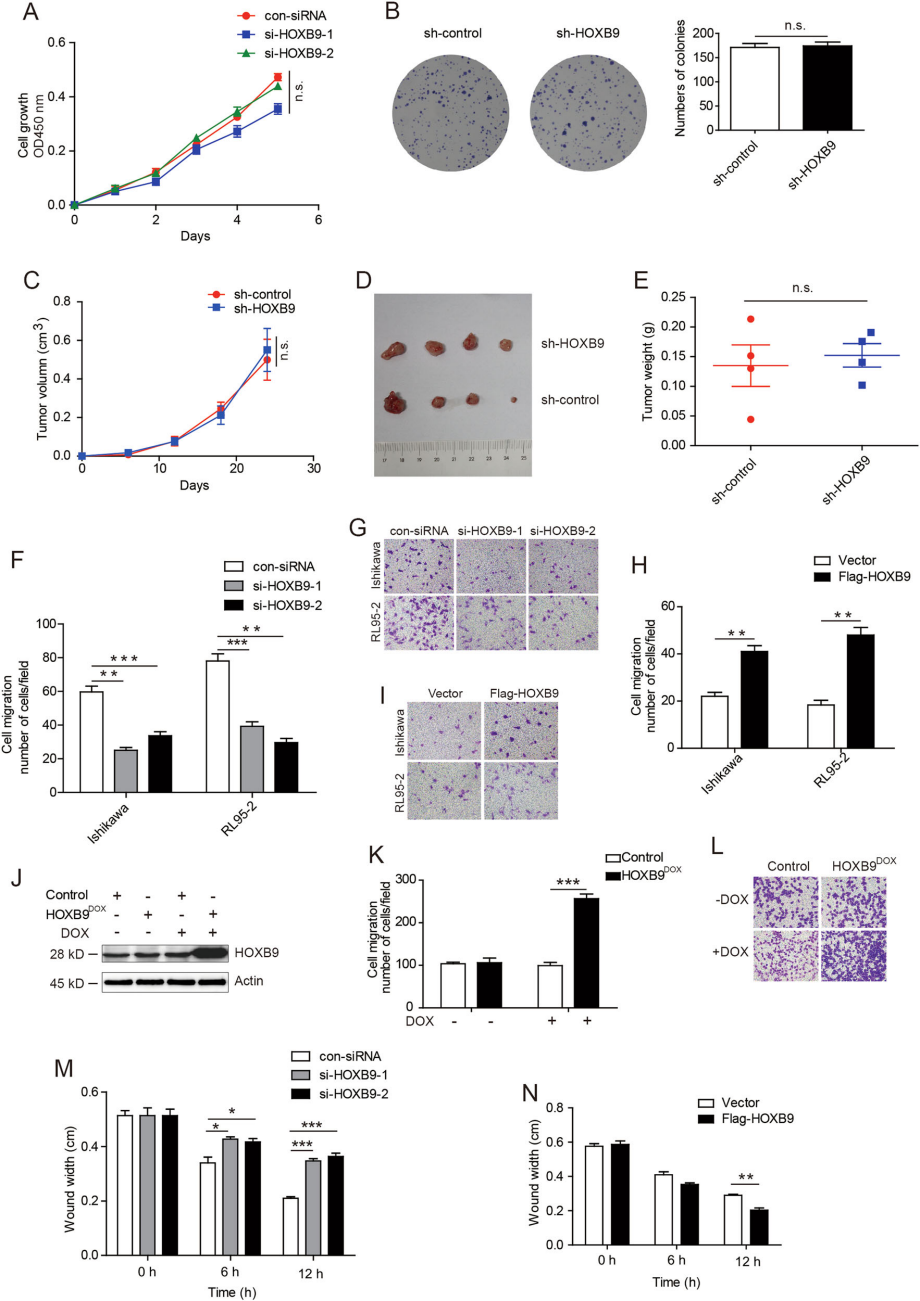
HOXB9 promotes endometrial cancer cell migration. **a** Cell proliferation assays were performed in endometrial cancer cell line Ishikawa, which was knocked down with two different HOXB9 small interfering RNA (siRNA). **b** The colony formation assays were performed using the Ishikawa stable cell lines infected with lentiviruses carrying control shRNA or HOXB9 shRNA. Cells were incubated for 2 weeks, and washed three times with PBS and fixed with 4% paraformaldehyde, following with staining by 0.5% crystal violet solution. **c** The mouse xenograft assays using the Ishikawa stable cell lines infected with lentiviruses carrying control shRNA or HOXB9 shRNA. Tumor growths in xenografted nude mice were measured and plotted. **d** The xenograft tumors were dissected and photographed at day 24**. e** Average tumor weights were measured at day 24. **f-h** The effect of HOXB9 on cell migration in Ishikawa and RL95-2 was determined. Data were presented as mean ± SEM from three independent experiments. Representative images of cell migration in Ishikawa and RL95-2 were shown. **j** Western blot was performed to detect the expression of HOXB9 by adding doxycycline. **k** The transwell assays were performed by adding doxycycline to induce HOXB9 expression. Data were presented as mean ± SEM from three independent experiments. **l** Representative images of cell migration were shown. **m, n** The wound-healing assays were performed in Ishikawa cells with knocked down or overexpressed of HOXB9, and images were taken at 0, 6 and 12 h after wound. The wound widths were measured and quantified. *P < 0.05, **P < 0.01, ***P < 0.001

Given the high expression level of HOXB9 in EC, we first investigated the effect of HOXB9 on cancer cell proliferation. However, when endogenous HOXB9 in EC cell line Ishikawa was knocked down with two different HOXB9 small interfering RNA (siRNA), there was no difference in the cell proliferation rate in the three groups (Fig. 2a). Then, we performed colony formation assays using the Ishikawa stable cell lines infected with lentiviruses carrying control short hairpin RNA (shRNA) or HOXB9 shRNA. However, the colony formation assays also showed that the depletion of HOXB9 in Ishikawa cells resulted in no significant decrease in the colony number (Fig. 2b). Moreover, mouse xenograft assays using the Ishikawa stable cell lines infected with lentiviruses carrying control shRNA or HOXB9 shRNA showed that cells expressing sh-HOXB9 displayed no significance in tumor growth rate and tumor volumes than that of the control group (Figs. 2c-e). It indicated that HOXB9 did not influence the proliferation ability of EC cell.

We then tested the EC cell migration mediated by HOXB9. Using transwell assays, we found that cell migration ability was decreased when knocked down HOXB9 in Ishikawa and RL95-2 cells (Figs. 2f, g). In contrast, after overexpression of HOXB9 in the two cancer cells, the cell migration ability markedly increased (Figs. 2h, i). To extend the study of the precise functional role of HOXB9 changed in vivo in regulating EC progression, we introduced a doxycycline (Dox)-inducible system, which allows the induction of HOXB9 by the addition of Dox in EC cell line Ishikawa. Western blot analysis revealed the expression of HOXB9 was markedly increased by adding Dox (Fig. 2j). Using transwell assays, we demonstrated that Dox-induced HOXB9 expression significantly increased cell migration capacity compared with the controls ± Dox or HOXB9-inducible cells without Dox induction (Figs. 2k, l). In addition, we performed wound-healing assays to determine whether HOXB9 affects the cancer cell migration. Similarly to the previous report, knocked down HOXB9 inhibited Ishikawa cell migration, and overexpression of HOXB9 accelerated migration of Ishikawa cell (Figs. 2m, n). Thus, these data suggested that HOXB9 mainly affect the EC cell migration, not cell proliferation.

### The expression between HOXB9 and E2F3 is positively correlated

**Fig. 3:**
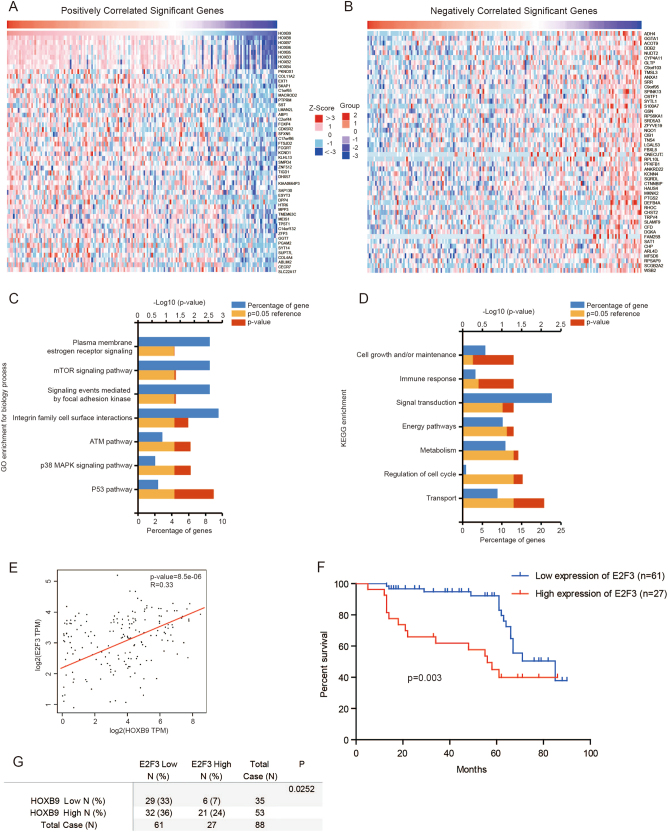
The expression between HOXB9 and E2F3 is positively correlated. **a, b** Genes highly co-expressed with HOXB9 in TCGA from the LinkedOmics database (http://www.linkedomics.org) were selected, and the top 50 positively **a** and 50 negatively **b** correlated genes are shown. **c, d** Gene ontology (GO) and Kyoto Encyclopedia of Genes and Genomes (KEGG) enrichment analysis on the correlated expressed genes. **e** Pearson correlation analysis revealed that E2F3 were significantly correlated with HOXB9 in human endometrial cancer using TCGA database. **f** The Kaplan–Meier analysis of E2F3 expression in 88 endometrial carcinoma was performed. **g** The correlation of HOXB9 and E2F3 expression in 88 endometrial carcinoma were analyzed

To investigate the signal pathways regulated by HOXB9 in EC, we analyzed series of genes that have strong co-expression correlation (Pearson *r* value > 0.2 or < –0.2) with HOXB9 in TCGA from the LinkedOmics database (http://www.linkedomics.org) (Figs. 3a, b). Next, we performed Gene ontology (GO) and Kyoto Encyclopedia of Genes and Genomes (KEGG) enrichment analysis on the correlated expressed genes. The enrichment analysis of GO biological process revealed that these genes were involved in a variety of processes, including P53, p38 Mitogen-activated Protein Kinase (MAPK),and Ataxia Telangiectasia Mutated Kinase (ATM) and integrin family cell surface interaction pathways (Fig. 3c). Meanwhile, KEGG enrichment analysis demonstrated that these genes were closely related to multiple pathways, including transport, regulation of cell cycle, metabolism and energy pathways (Fig. 3d).

**Fig. 4:**
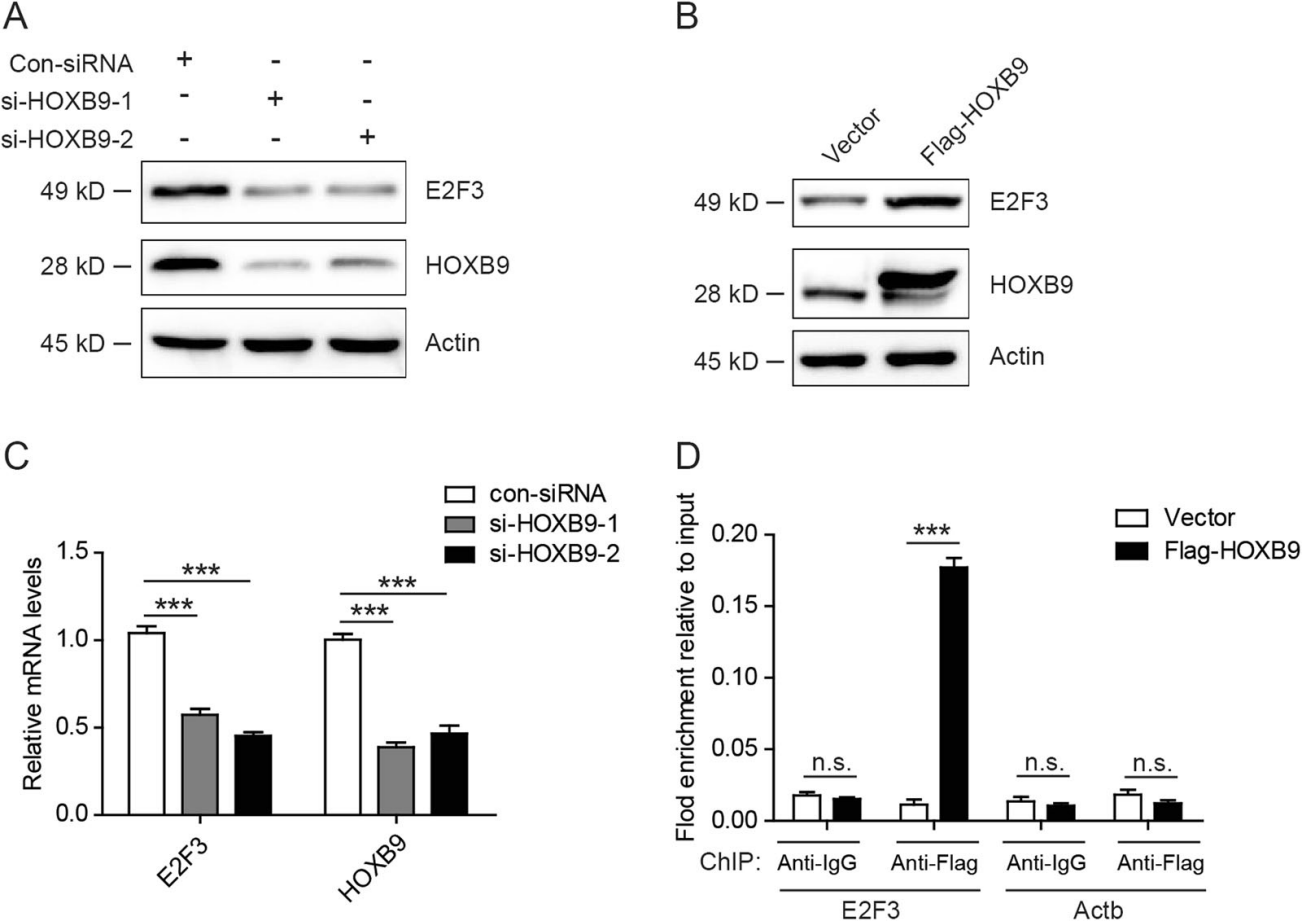
E2F3 is a direct downstream target of HOXB9. **a** The endometrial cancer cell line Ishikawa was knocked down with two different HOXB9 small interfering RNA (siRNA), and cell lysates were analyzed by western blot with the indicated antibodies. **b** The endometrial cancer cell line Ishikawa was transfected with Flag-HOXB9 or empty vector plasmid, and cell lysates were analyzed by western blot with the indicated antibodies. **c** The endometrial cancer cell line Ishikawa was knocked down with two different HOXB9 siRNA, and E2F3 and HOXB9 mRNA expression levels were analyzed by real-time PCR. **d** ChIP assays were performed using control IgG or anti-Flag antibody in Ishikawa cells. The binding of HOXB9 on promoters of E2F3 and the negative control Actb genes were analyzed by real-time PCR. Data were presented as mean ± SEM from three independent experiments. The statistical analyses were performed by Student’s *t-*test, ***P < 0.001

Among the total positively or negatively correlated significant genes, E2F3 was noted to act as oncogenic protein^33–35^. E2F3 was reported overexpressed in multiple tumors, and correlated with cancer metastasis. When we examined the mRNA expression levels of several tumor metastasis-related genes after knocking down HOXB9 (data not shown), we only found E2F3 mRNA expression levels were decreased significantly (Fig. 4c). Then, we analyzed the correlation of HOXB9 and E2F3 expression in TCGA EC database and found a positive correlation between HOXB9 and E2F3 mRNA expression levels (*R* = 0.33, *P* = 8.5e-06; Fig. 3e). In addition, the mRNA expression level of E2F3 was higher in EC than the normal tissues (Fig. S1d), and the high expression level of E2F3 correlated with the poor outcome of EC patients (Fig. S1e). To further clarify the expression of E2F3 in EC, we analyzed its expression in a series of 88 endometrial carcinoma using immunohistochemical staining. The staining scores of E2F3 expression level were dichotomized into two groups, low and high (Fig. S1f). Interestingly, we also found high E2F3 expression level correlated significantly with shorter survival time (*P* = 0.003; Fig. 3f). Meanwhile, we analyzed the correlation of HOXB9 and E2F3 expression in 88 endometrial carcinoma. Consistent with the result in TCGA database, we found the expression levels of HOXB9 was correlated with E2F3 expression (*P* = 0.0252; Fig. 3g). These results indicated that E2F3 indeed acts as an oncogenic protein in EC.

### E2F3 is a direct downstream target of HOXB9

Interestingly, we discovered that knocked down HOXB9 by two different siRNA in Ishikawa cells inhibited the protein expression of E2F3, and overexpression of HOXB9 increased protein levels of E2F3 (Figs. 4a, b). In addition, E2F3 mRNA expression levels were decreased when knocked down HOXB9 (Fig. 4c), indicating that E2F3 may be a transcriptional target of HOXB9. To investigate whether E2F3 is a direct target gene of HOXB9, we performed chromatin immunoprecipitation (ChIP) assays. Interestingly, the results showed that HOXB9 binds directly to the promoter of E2F3 (Fig. 4d). Moreover, we extracted the protein in the mice tumor in Fig. 2d, and analyzed the protein levels by western blot with the indicated antibodies. Consistently, both HOXB9 and E2F3 protein levels were decreased in the tumor with lentiviruses carrying HOXB9 shRNA, compared with control shRNA (Fig. S1g). Therefore, these results indicated that E2F3 is a direct downstream target of HOXB9.

### Knocking down E2F3 abolished the ability of HOXB9 in enhancing cancer cell migration

**Fig. 5:**
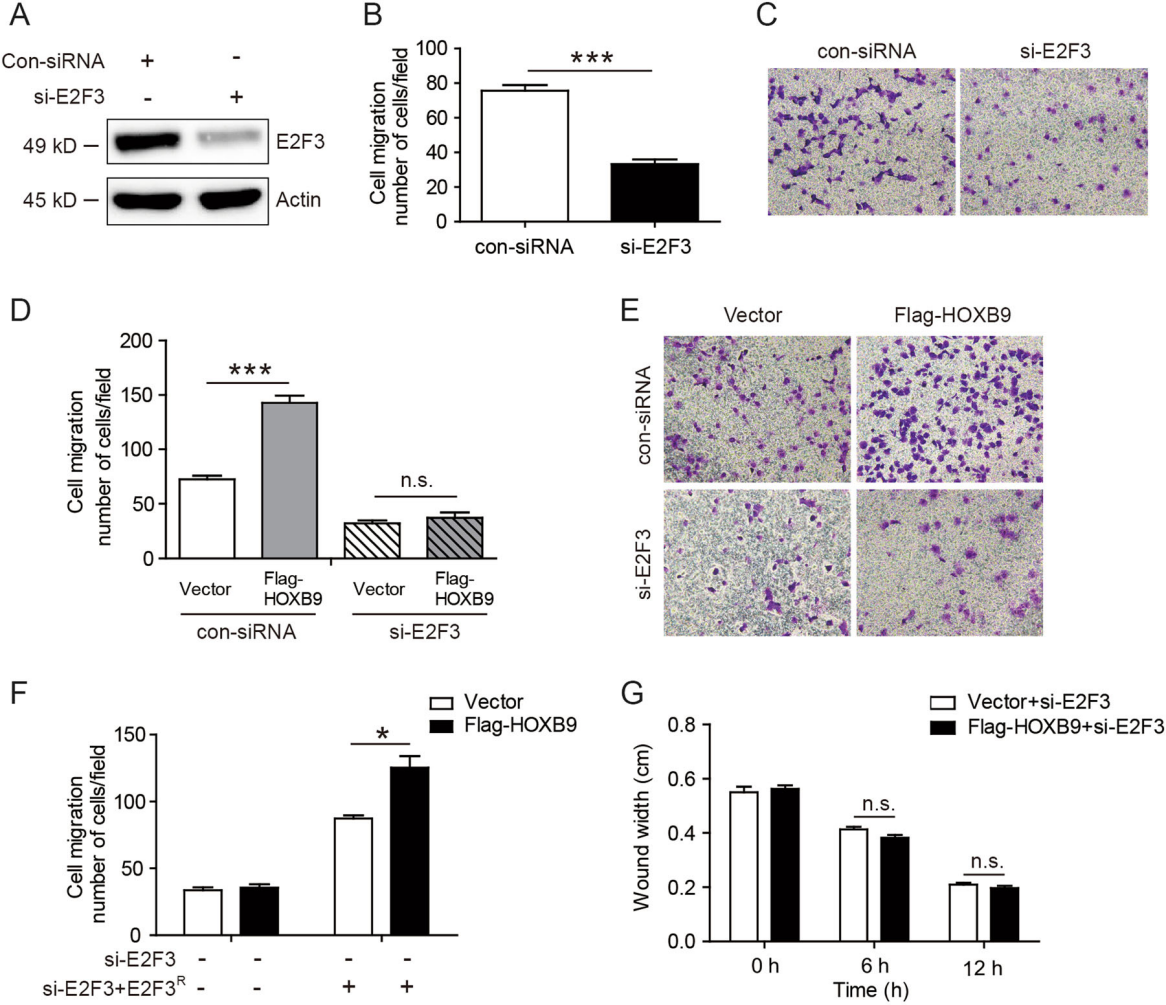
Knocking down E2F3 abolished the ability of HOXB9 in enhancing cancer cell migration. **a-c** Ishikawa was knocked down with E2F3 small interfering RNA (siRNA) **a**, and the effect of E2F3 on cell migration in Ishikawa was determined **b**. Data were presented as mean ± SEM from three independent experiments. The representative images of cell migration in Ishikawa were shown at the right panel **c**. **d, e** Ishikawa was firstly transfected with Flag-HOXB9, then knocked down with E2F3 siRNA, and the cell migration in Ishikawa was determined **d**. The representative images of cell migration in Ishikawa were shown at the right panel **e**. **f** We established the Ishikawa stable cell lines infected with lentiviruses carrying E2F3 shRNA. Next, we overexpressed E2F3 shRNA-resistant mutant plasmid in sh-E2F3 cells with the recovery of the E2F3 expression. Then, we overexpressed HOXB9 to detect the cell migration using transwell assays. **g** Ishikawa was first transfected with Flag-HOXB9, then knocked down with E2F3 siRNA, followed with wound-healing assays, and images were taken at 0, 6 and 12 h after wound. The wound widths were measured and quantified. The statistical analyses were performed by Student’s t-test, *<0.05, ***P < 0.001

To further elucidate the role of HOXB9 in regulating EC cell migration mediated by E2F3, we inhibited endogenous E2F3 in EC cell line Ishikawa with E2F3 siRNA (Fig. 5a). The transwell assays showed that knocked down E2F3 caused a significant decrease of Ishikawa cell migration (Figs. 5b, c). Furthermore, we found that knocking down E2F3 abolished the ability of HOXB9 in enhancing Ishikawa cell migration (Figs. 5d, e). Moreover, we established the Ishikawa stable cell lines infected with lentiviruses carrying E2F3 shRNA. However, using transwell assays, we found overexpression of HOXB9 did not increased cell migration ability in sh-E2F3 cells (Fig. 5f). Next, we overexpressed E2F3 shRNA resistant mutant plasmid in sh-E2F3 cells with the recovery of the E2F3 expression. Interestingly, we found that overexpression of E2F3 shRNA-resistant mutant plasmid increased cell migration ability. Surprisingly, after overexpression of HOXB9 in the cells with recovery of E2F3 expression, the cell migration ability markedly increased, compared with the vector group (Fig. 5f). In addition, the wound-healing assay results indicated that, after inhibiting E2F3, overexpressing HOXB9 did not affect the Ishikawa cell migration (Fig. 5g). Collectively, these findings showed E2F3 is an important downstream effector of HOXB9 in regulating EC cell migration.

## Discussion

EC is one of the most common gynecologic malignancy in the whole world^1,2,5^. From 1987 to 2008, the incidence of EC increased 50%, and the number of associated deaths increased approximately 300%. In 2017, an estimated 61,380 women in the United States were diagnosed with EC, and almost 11,000 died from this disease. Although there have been some chemotherapeutic and targeted therapy agents treated for ovarian, fallopian tube and primary peritoneal cancers, little therapy agents were effective for the palliative treatment of advanced EC^36,37^. Therefore, this highlights the need for understanding the molecular and biochemical mechanisms in advanced, recurrent, metastatic EC, and to develop new therapeutic approaches. Although there have been reports clarifying some signaling pathways in EC^38–43^, there were no reports about the relationship between HOXB9 and EC.

HOXB9 belongs to HOX family member. It mainly functions as a transcription factor, which plays important roles in embryo development and cancer progression^17,19–21,44^. The role of HOXB9 in cancer progression is complicated^15,16,45,46^. HOXB9 was found to be overexpressed in lung adenocarcinoma and human breast cancer, whereas in colon adenocarcinoma, pancreatic ductal adenocarcinoma and gastric carcinoma patients, its expression is decreased^15–19,21^. In this study, we found the expression of HOXB9 in endometrial carcinoma is higher than normal proliferative endometrium and atypical endometrial hyperplasia. In addition, HOXB9 expression notably correlated with histological grade and lymph node metastasis status. The results using immunohistochemical staining and bioinformatics analysis in TCGA EC database showed that elevated HOXB9 expression level corresponds to a poor overall survival in EC. Knocking down with HOXB9 siRNA only inhibits EC cell migration, but not cell proliferation, which partially explained why HOXB9 plays oncogenic role in EC progression.

HOXB9 was previously reported to regulate cancer progression, by targeting EMT via the transforming growth factor-β1/Smad2/Slug signaling pathway^47^. We also found HOXB9 regulated lung adenocarcinoma progression by targeting oncogenic protein JMJD6^18^. However, the mechanism underlying HOXB9 in EC progression remains unknown. Using bioinformatics analysis in TCGA EC database, we found many genes co-expressed with HOXB9. In addition, GO and KEGG analysis indicated these genes participated in multiple cancer-related signaling pathways. We picked the tumor-related protein E2F3. We found a positive correlation between HOXB9 and E2F3 mRNA expression levels. What’s more, knocked down HOXB9 inhibited the expression of E2F3 both in protein and mRNA levels. The ChIP assay showed E2F3 is a direct downstream target of HOXB9. In transwell and wound-healing assays, knocking down E2F3 abolished the ability of HOXB9 in enhancing cancer cell migration. These findings showed E2F3 is an important downstream effector of HOXB9 in regulating EC cell migration.

In summary, we have demonstrated that HOXB9 expression is increased in EC patients, and HOXB9 promotes EC cell migration by regulating E2F3 expression. Thus, HOXB9 may represent a prognostic marker and a potential therapeutic target for EC. However, the generic role of HOXB9 in vivo should be investigated in the future using transgenic mice models of HOXB9.

## Materials and methods

### Cell culture

Human EC cell lines, Ishikawa and RL95-2, were purchased from ATCC (American Type Culture Collection). All cells were grown in Dulbecco’s modified Eagle’s medium supplemented with 10% fetal bovine serum (FBS) and cultured at 37 °C with 5% CO_2_.

### Construction and antibodies

Flag-HOXB9 expression plasmid was constructed by subcloning the HOXB9 full-length complementary DNA (cDNA) fragments into 3× flag vector. All constructs were confirmed by DNA sequencing. The following antibodies were used: HOXB9 (Santa Cruz: sc-398500 and sc-133671) and E2F3 (Abcam: ab50917).

### Cell proliferation assays

Cell proliferation assays were performed using the CellTiter 96®AQueous One Solution Cell Proliferation Assay kit according to manufacturer’s instructions (Promega). Briefly, EC cells were plated in 96-well plates at a density of 900 cells per well. In all, 15 μl CellTiter 96®AQueous One Solution reagent was added to the cells per well, and incubated for 1 h at 37 °C. Then cell growth was measured in a microplate reader at 490 nm.

### Colony formation assay

Colony formation assay was used to determine the function of HOXB9 in promoting cell proliferation. In brief, 2000 cells were seeded onto six-well culture plates. After incubation for 2 weeks, cells were washed three times with phosphate-buffered saline (PBS) and fixed with 4% paraformaldehyde, following with staining by 0.5% crystal violet solution. The colonies were counted using a light microscope.

### In vivo xenograft tumor growth experiments

Balb/c nude mice was injected subcutaneously into the flank with 1 × 10^6^ Ishikawa stable cells. Tumor sizes were measured at the indicated time. After 24 days when the tumor reached to approximately 1 cm in diameter, the tumors were dissected, and the tumor weight was measured.

### Cell migration assays

Cell suspension containing 1 × 10^5^ cells/ml was seed into the upper chamber in serum-free media. The lower wells contains 20% (v/v) FBS. After 8-h migration at 37 °C, cells on the upper surface of the membrane filter were removed. The migrated cells through the inserts were fixed with 4% formaldehyde and stained by crystal violet, and counted.

### Wound-healing assays

The EC cell lines were cultured until a monolayer forms. Artificial wounds were created on the cell monolayer, then capture the images of wound healing at 0, 6 and 12 h, and compare the images to quantify the cell migration rate.

### Real-time PCR

Total RNA was isolated from cells using Trizol reagent (Invitrogen) and cDNA was synthesized. Two-step real-time polymerase chain reaction (PCR) was performed using the SYBR Green Mix (Roche) and a LightCycler®96 detection system (Roche) according to manufacturer’s instructions. Specific primers for HOXB9 and E2F3 were designed as follows:

*HOXB9* forward primer, 5ʹ-CCATTTCTGGGACGCTTAGCA-3ʹ; *HOXB9* reverse primer, 5ʹ-TGTAAGGGTGGTAGACGGACG-3ʹ; *E2F3* forward primer, 5ʹ-AAGAAGAAGTCTAAAAACAACGTCCAA-3ʹ; *E2F3* reverse primer, 5ʹ-CTTGACACTGGGCCAGCAT-3ʹ.

All mRNAs were normalized to the mRNA level of *GAPDH* gene.

### ChIP assays

The EC cell lines were crosslinked with 1% formaldehyde for 10 min, and subjected to ChIP assays as described before^48^. The ChIP DNA complex were precipitated and subjected to RT-PCR with the indicated primers.

### Bioinformatics analysis of TCGA database

The results of TCGA database were mainly analyzed by the web-based tools, GEPIA (http://gepia.cancer-pku.cn/)^31^ and the LinkedOmics database (http://www.linkedomics.org)^32^, which are web-based tools to deliver fast and customizable functionalities based on TCGA data. GEPIA can deliver fast and customizable functionalities based on TCGA data, and provides key interactive and customizable functions including differential expression analysis, correlation analysis and patient survival analysis. The LinkedOmics web allows flexible exploration of associations between a molecular or clinical attribute of interest and all other attributes, providing the opportunity to analyze and visualize associations between billions of attribute pairs for each cancer cohort.

### Immunohistochemical staining

The endometrial carcinoma were obtained from surgical excision in the third affiliated hospital of Zhengzhou University. According to age, the endometrial carcinoma were divided into: ≤50, 41 cases; >50, 47 cases. Surgical staging: stage I, 53 cases; stage II, 18 cases; stage III–IV, 17 cases. Histological grade: G1, 29 cases; G2, 26 cases; G3, 33 cases. Muscular invasion endomembrane: ≤1/2, 39 cases; >1/2, 49 cases. Lymph node metastisis: Yes, 39 cases; No, 49 cases. Normal endometrial samples were obtained from premenopausal women awaiting in vitro fertilization treatment. All of the above cases were treated for the first time. No chemotherapy, radiotherapy and other adjuvant treatment had been done before the operation. The atypical endometrial hyperplasia was characterized by numerous glands with irregular contour, papillary intraglandular proliferations, stratified epithelium (2–4 lines), with the loss of polarity and marked nuclear atypia, also presenting atypical mitoses. The glands were extremely irregular with respect to shape and size, arranged “back-to-back”. However, the endometrial carcinoma were characterized by a glandular proliferation marked with intra-luminal papillary projections, with the presence of outgrowths and branches that realize a confluent pattern. These are lined by stratified neoplastic epithelia with a thin fibrovascular axis.

Immunohistochemical staining was performed using 5-mmol/L thick sections. Tissue sections were deparaffinized and rehydrated gradually, and antigen retrieval was performed in pH 6.0 citrate buffer for 10 min. To quench endogenous peroxidases, peroxide blocking was performed for 30 min with 3% H_2_O_2_. The slides were incubated with primary antibody at 4 °C overnight, then the two-step plus poly-HRP anti-rabbit/mouse IgG detection system was applied. The immunohistochemical staining results were assigned a mean score considering both the intensity of staining and the proportion of cells with an unequivocal positive reaction. Each section was independently assessed by two pathologists without prior knowledge of patient data. Positive reactions were defined as those showing brown signals mainly in the cell nucleus. A staining index (values, 0–3) was determined by the staining intensity and positive area. The scores (0–3) were defined as follows: 0, negative; 1, weak; 2, moderate; and 3, strong. For statistical analysis, scores of 0–1 were considered low expression and scores of 2–3 considered high expression.

### Statistical analysis

Chi-square tests were used to determine category variable differences. Overall survival distributions were estimated using Kaplan–Meier analysis. For other experiments, statistical significance was determined by Student’s *t*-test.

## Electronic supplementary material

FigureS1

Supplementary Information
