# Supplementary figures and images for: HOXB9 promotes endometrial cancer progression by targeting E2F3
Source: Cell Death Dis. 2018 May 3;9(5):509. doi: 10.1038/s41419-018-0556-3 (PMC5938704; doi:10.1038/s41419-018-0556-3)

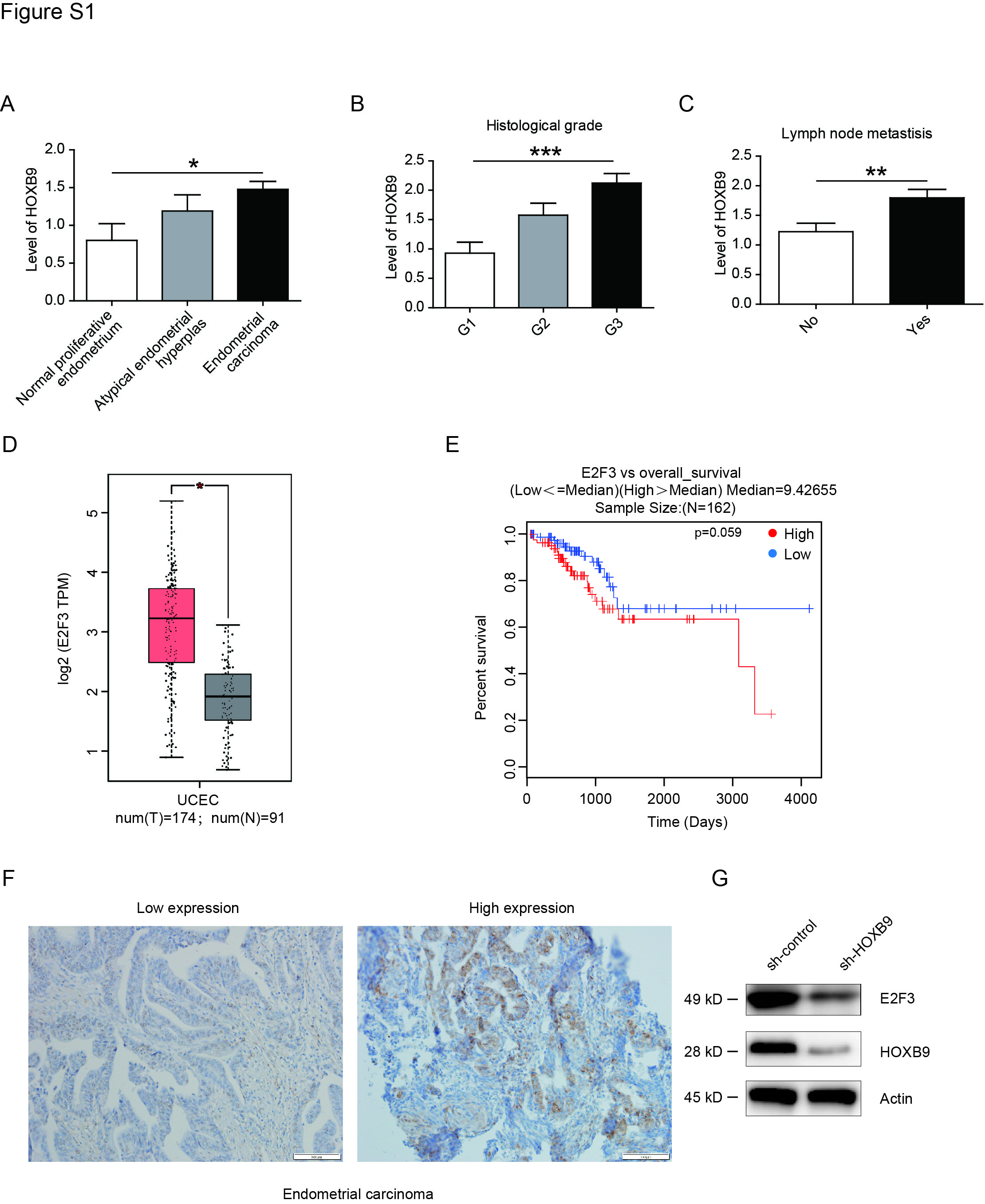

Supplement: Supplementary file 1 — FigureS1 [file 41419_2018_556_MOESM1_ESM.jpg]
