# Supplementary material for: HOXB9 promotes endometrial cancer progression by targeting E2F3
Source: Cell Death Dis. 2018 May 3;9(5):509. doi: 10.1038/s41419-018-0556-3 (PMC5938704; doi:10.1038/s41419-018-0556-3)
Supplement: Supplementary file 2 — Supplementary Information [file 41419_2018_556_MOESM2_ESM.docx]

**SUPPLEMENTARY DATA**

**Supplementary Figure legends**

**Fig. S1.** Expression levels of HOXB9 were compared between different tissue (**a**), histological grade (**b**) and lymph node metastasis status (**c**). **d-e** The expression level of E2F3 in human endometrial cancer compared with normal tissues in TCGA database **(d)**, and the Kaplan-Meier analysis of E2F3 expression in TCGA endometrial cancer database **(e)**. **f** The staining scores of E2F3 expression level were dichotomized into two groups, low (score of 0 or 1) and high (score of 2 or 3). We have displayed the representative images of ‘low’ and ‘high’ staining in endometrial carcinoma. **g** We extracted the protein in the mice tumor in Fig. 2d, and analyzed the protein levels by western blot with the indicated antibodies. The statistical analyses were performed by one way ANOVA analyses **(a-c)**, Student’s t-test **(d)** and Kaplan-Meier analysis **(e)**. * for P<0.05, ** for P<0.01, *** for P<0.001.
